# CONSEQUENCES OF AGE DISCRIMINATION IN THE WORKPLACE

**DOI:** 10.1093/geroni/igz038.473

**Published:** 2019-11-08

**Authors:** Dannii Yeung

**Affiliations:** City University of Hong Kong, Hong Kong, Hong Kong

## Abstract

Despite a growing number of middle-aged employees in the workforce, Hong Kong still lags behind other developed countries in implementing age-friendly policies to protect older workers from being discriminated in recruitment, performance appraisal, and other personnel decisions. This paper therefore aims to investigate the effects of age discrimination on work-related outcomes and well-being. A total of 333 Hong Kong Chinese employees aged 40 years and above (Mage = 46.6 years, SD = 6.21, Range = 40-68; 60% female) completed an online survey on work experiences. Self-perceived age discrimination in the workplace (Furunes & Mykletun, 2010), perception of occupational future time (Ho & Yeung, 2017; Zacher & Frese, 2009), work engagement (Schaufeli et al., 2017), work stress (Cohen et al., 1983), and psychological distress (Shek, 1989) were measured. Almost 53% of the participants reported that older workers were less likely to get the same training opportunity for new technology as their younger peers, and 46.8% perceived that older workers did not get the same promotion opportunity as their younger peers. Regression analyses further revealed that employees’ perceived age discrimination in the workplace indirectly influenced their levels of work engagement (B = -.038, SE = .017), work stress (B = .016, SE = .005), and psychological distress (B = .008, SE = .005) through perception of occupational future time. The findings of this study unveil the severity of workplace age discrimination in Hong Kong and the negative impacts of age discrimination on work-related outcomes. Recommendations for organizational practices will be discussed.

